# The association between erosive tooth wear and diet, hygiene habits and health awareness in adolescents aged 15 in Poland

**DOI:** 10.1007/s40368-021-00670-x

**Published:** 2021-10-12

**Authors:** E. Rusyan, E. Grabowska, I. Strużycka

**Affiliations:** 1 Department of Conservative Dentistry, Warsaw Medical University, Warsaw, Mazowieckie Poland; 2 Department of Periodontology and Oral Diseases, Warsaw Medical University, Warsaw, Mazowieckie Poland; 3 Department of Integrated Dentistry, Warsaw Medical University, Warsaw, Mazowieckie Poland

**Keywords:** Erosive tooth wear (ETW), Epidemiologic studies, Risk factors, Basic Erosive Wear Examination (BEWE)

## Abstract

**Purpose:**

The aim of the study was to assess the prevalence of erosive tooth wear (ETW) and risk indicators in the population of adolescents aged 15 in Poland.

**Methods:**

Erosive tooth wear in 2639 participants was determined by calibrated examiners according to the BEWE scoring system, and the prevalence of risk factors was assessed on the basis of a survey.

**Results:**

Erosive tooth wear was reported in 24.3% of participants. Initial loss of surface (BEWE 1) was the predominant finding, observed in 21.3% of participants. Hard tissue loss (BEWE 2 and 3) occurred very rarely, only in 3% of participants. Acidic diet, masculine gender and lower socio-economic status were associated with higher prevalence and severity of erosive lesions in the examined population.

**Conclusion:**

Two modifiable factors—acidic diet and low health awareness—were found to be highly unsatisfactory in the adolescents aged 15 in Poland. Accordingly, to prevent the deterioration of the functionality and aesthetics of the teeth in young people, certain measures, such as routine clinical examination, education, dietary consulting and prophylaxis, should be implemented as early as possible, focusing predominantly on families with lower socio-economic status.

## Introduction

Erosive tooth wear (ETW) in children and adolescents is a widespread phenomenon these days. Epidemiological studies on the occurrence of erosion wear conducted in European countries clearly show that the problem applies to all age groups; however, a particular increase in the frequency is observed among adolescents and young adults (Bartlett et al. 2013; Jaeggi and Lussi 2014; Provatenou et al. 2016).

Changes in lifestyle, eating patterns and hygiene, studied in recent years, increase the number of patients diagnosed with non-carious dental hard tissue defects, including erosive wear. The consumption of acidic foods and beverages has increased considerably, both in quantity and frequency. Apart from dental hygiene habits and socioeconomic status, diet is the most frequently studied risk factor of the disease (Wang et al. 2010; Haifeng et al. 2012; Lussi et al. 2014; Okunseri et al. 2015; Skalsky et al. 2017).

The aim of this study was to assess the incidence of ETW in adolescents and to identify risk factors potentially related to the development of the disease. The authors attempted also to assess the level of health awareness of adolescents in relation to erosive lesions.

## Materials and methods

The clinical study on the occurrence of ETW in the population of 15-year-old adolescents was carried out as part of the National Program of Oral Health Monitoring. This program is conducted in cooperation with the Ministry of Health and the Medical University of Warsaw.

**Fig.1:**
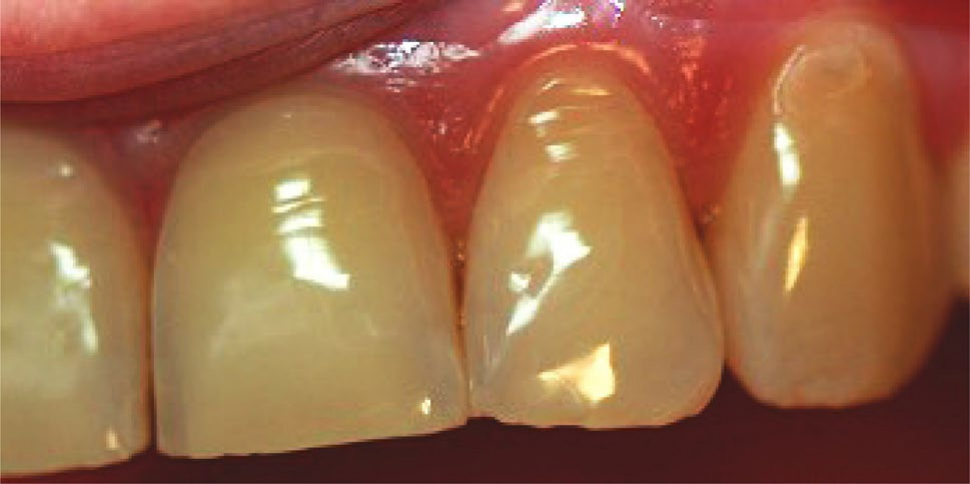
Severe erosive tooth wear on the facial surfaces of anterior teeth. Age of patients: 15 years

**Fig. 2:**
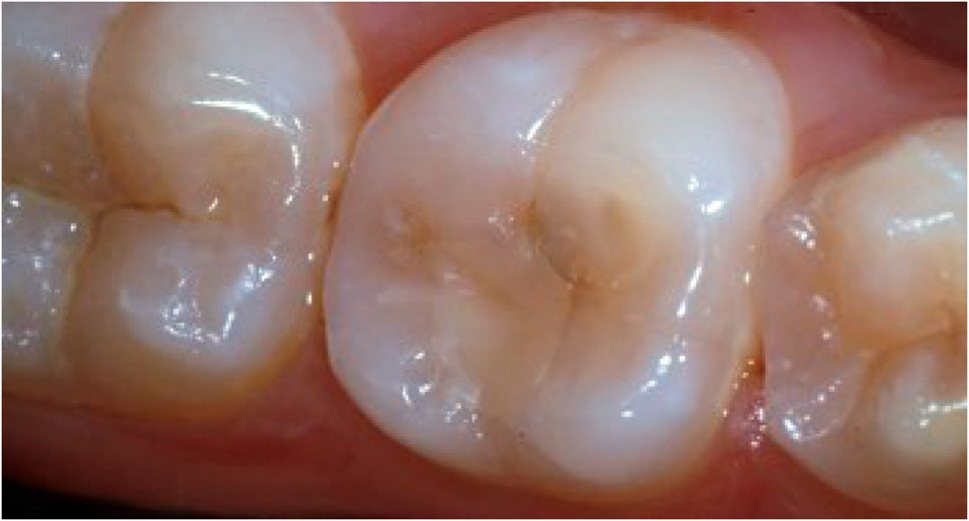
A 15-year-old adolescent with severe erosive lesions affecting the permanent molars

**Fig. 3:**
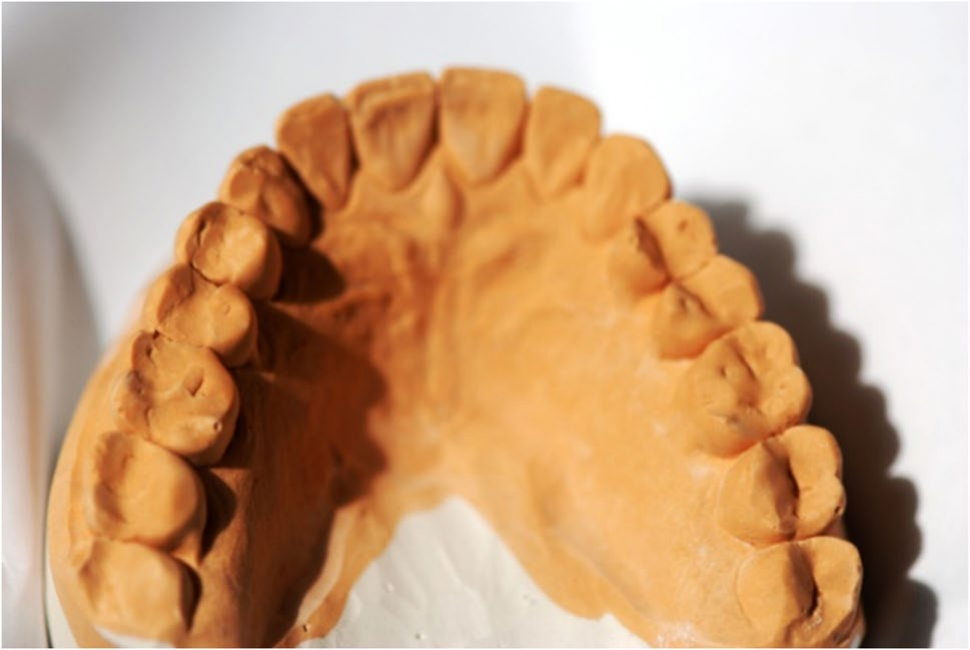
The plaster model of a patient with eating disorders, advanced erosive tooth wear

The study group was selected by stratified sampling and included the inhabitants of nine (out of 16) provinces of Poland. In each province, the research was carried out in two counties, one urban and one rural, from which schools educating young people in a set age group were randomly selected. The study included 2639 participants, of whom 1368 were girls and 1271 were boys, who participated in an ordinary checkup study, not related to emergency intervention. Provinces were selected on the basis of regional division, ensuring that the survey was conducted in all parts of the country. Two dentists conducted the study in each county, for a total of 18 investigators involved in the study. All investigators received lecture-based training along with ETW evaluation on photographs and plaster models, examples of which are shown in Figs. 1, 2, 3, combined with a calibration process. Calibration was based on examination of a group of 26 adolescents with and without ETW by the study team and by experienced epidemiologists (IS and ER) with known high reproducibility of clinical assessments. The investigators conducted the study two times, 1 week apart. By comparing the results of the consecutive examinations, the magnitude of the inter-examiner reliability error and the repeatability of each individual's assessment was determined when an investigator performed a repeat examination on the same patient.

Accuracy and reproducibility of at least 85% (Kappa Statistics) were registered in all surveys.

Both parents and children received written and oral information about the study, and written consent was obtained from parents of adolescents for the participation in the study.

Cases of refusal to participate in the study were sporadic; however, one of the middle school classes drawn as a whole refused to participate in the study. A part of the examined adolescents, fearing the loss of anonymity, did not agree to mark the questionnaire and examination card with the same number. These cases were excluded from the study. In total, the clinical and sociomedical examination covered 83% of the drawn sample.

The teeth were examined in the conditions of a dental office under artificial lighting. All tooth surfaces were examined for erosive wear based on the criteria of the “Basic Erosive Wear Examination” indicator (BEWE; Code 0: no erosive tooth wear; Code 1: initial loss of surface texture; Code 2: distinct defect, hard tissue loss < 50% of the surface area; Code 3: hard tissue loss ≥ 50% of the surface area) (Bartlett et al. 2008).

The dental examination was preceded by a socio-medical assessment, identifying potential risk factors for the occurrence of erosive lesions. The questions in the survey included: demographic data (gender, place of residence, parents’ education, parents’ working status), general health (allergies, asthma, gastroesophageal reflux and eating disorders); health-related behaviors (type of toothbrush used, time between meal and toothbrushing, fluoride use); and perceived tooth sensitivity. The participants were also asked to assess the validity of three basic statements regarding the definition of tooth erosion as well as the importance of acidic diet and over-vigorous toothbrushing in ETW etiology. Moreover, the frequency of consumption of acidic foods and beverages by the study participants was assessed.

Data analyses were performed using PQStat software (PQStat v. 1.4.4). Descriptive statistics were presented as number and percentage of participants or as a median, mean and SD values. The χ^2^ test was used for testing the significance of differences between the general prevalence of ETW, as well as the moderate or severe lesions prevalence, depending on the potential risk factors. Odds ratio (with 95% confidence interval) for ETW presence was also presented for each of the factors. The Mann–Whitney test was used to compare the frequency of acidic foods and drinks consumption between boys and girls. The correlation between the severity of ETW and potential risk factors, as well as between the BEWE values in anterior and posterior teeth, was tested by means of Spearman rank correlation test. A *P* value < 0.05 was considered statistically significant.

The research was approved by the Bioethics Committee of the Medical University of Warsaw.

## Results

The general characteristic of the study group is presented in Table 1.

**Table 1:** General characteristics of the study group

Gender	Male: 1271 (48.2%) Female: 1368 (51.8%)
Place of residence	Urban area: 1542 (58.4%) Rural area: 1097 (41.6%)
Education level of the mother	Primary: 109 (4.1%) Secondary: 1715 (65.0%) University: 556 (21.1%) Missing data: 259 (9.8%)
Education level of the father	Primary: 116 (4.4%) Secondary: 1823 (69.1%) University: 374 (14.2%) Missing data: 326 (12.4%)
Working status of the mother	Full-time job: 1588 (60.2%) Part-time job: 287 (10.9%) Unemployed: 557 (21.1%) Missing data: 207 (7.8%)
Working status of the father	Full-time job: 1860 (70.5%) Part-time job: 269 (10.2%) Unemployed: 232 (8.8%) Missing data: 278 (10.5%)
Systemic health	Gastroesophageal reflux: 475 (18.4%) Eating disorders: 95 (3.7%) Allergies: 20 (0.8%) Asthma: 11 (0.4%)
Toothbrush type	Manual, soft: 145 (5.5%) Manual, medium: 1672 (63.4%) Manual, hard: 438 (16.6%) Electric: 256 (9.7%) No toothbrush and/or missing data: 360 (13.6%)
Time between meal and toothbrushing	Median: 5 min Mean: 16 min, SD = 37 min
Fluoride rinse or gel	1757 (66.6%)
Tooth sensitivity	613 (23.2%)

The overall percentage of participants presenting signs of ETW in the studied population was 24.3% (Table 2). Erosive lesions occurred most often in the form of initial loss of enamel surface texture (BEWE 1: 21.3% of the studied sample). More advanced changes (BEWE 2 and 3) were observed very rarely (3% of the studied sample).

**Table 2:** The maximum values of the BEWE index in persons aged 15

	BEWE = 0	BEWE = 1	BEWE = 2	BEWE = 3	Spearman rank correlation
Total	1997 75.7%	563 21.3%	74 2.8%	5 0.2%	–
Anterior teeth	2158 81.8%	433 16.4%	44 1.7%	4 0.2%	*r* = 0.37 *P* < 0.0001*
Posterior teeth	2373 89.9%	231 8.8%	34 1.3%	1 < 0.1%	

Significance level *p < 0.05

Erosive lesions were more frequently located in the anterior compared to the posterior teeth (18.2% and 10.1% prevalence, respectively). A moderately strong correlation (Spearman’s *r* = 0.37, *P* < 0.0001) between ETW severity in the anterior and the posterior teeth was observed.

The association between prevalence of ETW and related factors was also analyzed. No significant differences were found between the residents of urban and rural areas.

In the group of boys, the percentage of participants with erosive lesions in any of the sextants was slightly higher (26.2%) than in the group of girls (22.6%); the odds ratio for presence of erosive lesions in boys as compared to girls was 1.22 (95% CI 1.02–1.45; *P* = 0.03; Table 3). This difference was even more profound when more advanced erosive lesions were concerned (OR = 1.69; 95% CI 1.07–2.68; *P* = 0.02 for BEWE ≥ 2).

**Table 3:** Prevalence of ETW, depending on the presence of potential risk factors

		General erosive lesions prevalence (BEWE ≥ 1)	OR (95% CI) *P* value	Moderate or severe erosive lesions prevalence (BEWE ≥ 2)	OR (95% CI) *P* value
Place of residence	Urban area	23.7%	Ref	3.1%	Ref
	Rural area	25.3%	1.09 (0.91–1.30) *P* = 0.35	2.8%	0.91 (0.57–1.43) *P* = 0.67
Gender	Male	26.2%	1.22 (1.02–1.45) *P* = 0.03*	3.8%	1.69 (1.07–2.68) *P* = 0.02*
	Female	22.6%	ref	2.3%	ref
Girls, depending on the place of residence	Urban area	21.3%	ref	2.2%	ref
	Rural area	24.5%	1.19 (0.92–1.54) *P* = 0.17	2.4%	1.07 (0.52–2.20) *P* = 0.86
Boys, depending on the place of residence	Urban area	26.3%	1.01 (0.79–1.30) *P* = 0.92	4.1%	1.26 (0.70–2.29) *P* = 0.44
	Rural area	26.1%	Ref	3.3%	Ref
Education level of the mother	Primary	26.6%	1.08 (0.70–1.67) *P* = 0.73	1.8%	0.55 (0.13–2.30) *P* = 0.42
	Secondary	25.1%	Ref	3.3%	Ref
	University	22.7%	0.87 (0.70–1.09) *P* = 0.24	2.5%	0.77 (0.42–1.39) *P* = 0.38
Education level of the father	Primary	22.4%	0.86 (0.55–1.35) *P* = 0.52	3.4%	1.13 (0.40–3.16) *P* = 0.82
	Secondary	25.1%	Ref	3.1%	Ref
	University	21.7%	0.83 (0.63–1.08) *P* = 0.16	2.9%	0.96 (0.50–1.84) *P* = 0.89
Working status of the mother	Full-time job	25.7%	Ref	3.4%	Ref
	Part-time job	26.8%	1.06 (0.77–1.47) *P* = 0.72	6.3%	1.89 (0.98–3.67) *P* = 0.06
	Unemployed	23.9%	0.91 (0.73–1.14) *P* = 0.41	2.5%	0.73 (0.42–1.27) *P* = 0.27
Working status of the father	Full-time job	19.0%	Ref	4.3%	Ref
	Part-time job	25.3%	1.45 (0.94–2.22) *P* = 0.09	3.3%	0.77 (0.31–1.92) *P* = 0.57
	Unemployed	24.7%	1.40 (0.99–1.98) P = 0.06	3.0%	0.69 (0.35–1.37) P = 0.29
Systemic health	Gastroesophageal reflux	23.8%	0.94 (0.74–1.19) *P* = 0.60	3.4%	1.13 (0.65–1.97) *P* = 0.67
	Eating disorders	18.9%	0.70 (0.42–1.18) *P* = 0.19	2.1%	0.67 (0.16–2.78) *P* = 0.58
	Allergies	20.0%	0.76 (0.25–2.28) *P* = 0.62	5.0%	1.67 (0.22–12.65) *P* = 0.62
	Asthma	9.1%	0.30 (0.04–2.38) *P* = 0.26	0	–
Toothbrush type	Manual, soft	27.6%	1.17 (0.80–1.72) *P* = 0.41	3.4%	1.09 (0.43–2.77) *P* = 0.85
	Manual, medium	24.5%	Ref	3.2%	Ref
	Manual, hard	24.7%	1.01 (0.79–1.29) *P* = 0.95	3.0%	0.93 (0.50–1.73) *P* = 0.83
	Electric	27.0%	1.14 (0.84–1.53) *P* = 0.40	3.1%	0.99 (0.46–2.10) *P* = 0.97
Time between meal and toothbrushing	≤ 5 min	24.4%	0.96 (0.80–1.15) *P* = 0.65	3.0%	0.92 (0.58–1.44) P = 0,71
	> 5 min	25.2%	Ref	3.2%	Ref
Fluoride rinse or gel	Yes	25.1%	1.03 (0.85–1.25) *P* = 0.75	2.7%	0.82 (0.50–1.35) *P* = 0.44
	No	24.5%	Ref	3.2%	Ref
Tooth sensitivity	Yes	26.3%	1.09 (0.89–1.35) *P* = 0.39	4.9%	1.99 (1.25–3.17) *P* = 0.004*
	No	24.6%	Ref	2.5%	Ref

*ref.* reference level, significance level *p < 0.05

When analyzed separately, higher prevalence of ETW in girls was stated in the rural areas, although this difference was not significant. For boys, on the contrary, the ETW prevalence tended to be slightly higher in the urban areas.

Another factor related to the prevalence of erosive wear, including the more severe forms, was the working status of the parents. A father working part-time and/or being unemployed increased the risk of erosive tooth wear in children, as presented in Table 3. For mothers, this relation was not that clear, yet a slightly higher risk was stated if the mother was working part-time.

The declared tooth sensitivity was also related to significantly higher prevalence of moderate or severe erosive lesions (OR = 1.99; 95% CI 1.25–3.17). While it cannot be considered risk factor, it can serve as a screening question, indicating a need to perform particularly meticulous clinical examination for signs of ETW.

The most frequently consumed acidic products were: fruits, fruit juices and fruit teas (Table 4). Daily consumption of such products was declared by 74.2%, 64.5% and 49.9% of participants, respectively, while 17.8%, 25.1% and 15.2% consumed these groups of products at least three times a day. Fruit juices predominated among the products consumed at least six times a day (6.5%), followed by carbonated drinks (4.5%). Moreover, the consumption of fruit teas, isotonic, carbonated and energy drinks, as well as marinades was significantly higher among boys.

**Table 4:** Consumption of acidic foods and drinks in the study group

		Daily	≥ 3 times a day	≥ 6 times a day	Comparison (girls vs boys, Mann–Whitney test)
Consumption of acidic foods and drinks	Fruits	1958 (74.2%)	471 (17.8%)	80 (3.0%)	*P* = 0.64
	Fruit juices	1702 (64.4%)	662 (25.1%)	171 (6.5%)	*P* = 0.16
	Fruit teas	1318 (49.9%)	402 (15.2%)	95 (3.6%)	*P* = 0.03* (boys > girls)
	Isotonic drinks	282 (10.7%)	115 (4.4%)	42 (1.6%)	*P* < 0.001* (boys > girls)
	Carbonated drinks	996 (37.7%)	336 (12.7%)	118 (4.5%)	*P* < 0.001* (boys > girls)
	Energy drinks	225 (8.5%)	91 (3.4%)	30 (1.1%)	*P* < 0.001* (boys > girls)
	Marinades	128 (4.9%)	43 (1.6%)	22 (0.8%)	*P* = 0.008* (boys > girls)

Significance level *p < 0.05

The performed statistical analysis showed a positive, yet weak correlation between the erosive lesions of the anterior teeth and the consumption of carbonated and energy drinks (Spearman’s *r* = 0.05; *P* = 0.007 and *r* = 0.05; *P* = 0.02, respectively; Table 5).

**Table 5:** The correlation between the severity of ETW and the frequency of acidic foods or beverages consumption

Acidic foods and beverages	Spearman rank correlation	
	Erosive lesions in anterior teeth	Erosive lesions in posterior teeth
Fruits	*r* = 0.01 *P* = 0.79	*r* = 0.03 *P* = 0.21
Fruit juices	*r* = 0.02 *P* = 0.39	*r* = − 0.02 *P* = 0.42
Fruit teas	*r* = − 0.02 *P* = 0.37	*r* = − 0.03 *P* = 0.13
Isotonic drinks	*r* = 0.03 *P* = 0.13	*r* = 0.01 *P* = 0.77
Carbonated drinks	*r* = 0.05 *P* = 0.007*	*r* = 0.02 *P* = 0.37
Energy drinks	*r* = 0.05 *P* = 0.02*	*r* = 0.00 *P* = 0.95
Marinades	*r* = 0.00 *P* = 0.84	*r* = 0.01 *P* = 0.50

Significance level *p < 0.05

The last part of the questionnaire included three questions focused on the etiology of erosive lesions. The level of health awareness of the study participants was examined by analyzing the answers assigned to each statement (*true, false, I don’t know*). The last indication, *I don’t know*, was treated as synonymous with the subject’s lack of knowledge on the topic. The level of knowledge of the examined population about the erosive wear should be considered unsatisfactory (Table 6). Based on the analysis, it can be concluded that adolescents are more aware of the negative impact of hard toothbrushes and abrasive toothpastes on the condition of hard dental tissues than on the role of acidic diet in the development of erosive lesions.

**Table 6:** Health awareness related to ETW in the study group

		Distribution of answers in the study group		
		True	False	I don’t know
Health awareness related to tooth erosion	Erosion is caused by acids	470 (17.8%)	409 (15.5%)	1760 (66.7%)
	Fruits and fruit juices dissolve teeth	322 (12.2%)	1341 (50.8%)	976 (37.0%)
	Hard brushes and abrasive toothbrushes can damage teeth	1380 (52.3%)	313 (11.9%)	946 (35.8%)

The statistical analysis did not reveal any significant differences in the prevalence of erosive lesions, depending on the health awareness related to this topic (Table 7).

**Table 7:** The association between the prevalence of erosive lesions and the health awareness

		Erosive lesions in anterior teeth	OR (95% CI) *P* value	Erosive lesions in posterior teeth	OR (95% CI) *P* value
Erosion is caused by acids	True	17.4%	0.94 (0.72–1.22) *P* = 0.63	11.1%	0.79 (0.58–1.08) *P* = 0.14
	False/I don’t know	18.4%	Ref	13.6%	Ref
Fruits and fruit juices dissolve teeth	True	18.3%	1.01 (0.75–1.36) *P* = 0.96	13.4%	1.02 (0.72–1.44) *P* = 0.91
	False/I don’t know	18.2%	Ref	13.1%	Ref
Hard brushes and abrasive toothbrushes can damage teeth	True	19.1%	1.12 (0.92–1.37) *P* = 0.25	13.4%	1.05 (0.84–1.31) *P* = 0.68
	False/I don’t know	17.3%	Ref	12.9%	Ref

*ref.* reference level

## Discussion

The conducted research was an epidemiological study covering one of the WHO index age groups, which is 15-year-old adolescents. It is an important age, considered by some to be critical for shaping behaviors, including health awareness. The changes that take place in adolescence determine whether young people develop a positive potential or risk factors to their own and other people's health and carry them on into adulthood. The occurrence and intensity of ETW in the studied population of 15-year-olds is at a lower level than that described in the literature of the subject (Deery et al. 2000; Van Rijkom et al. 2002; Arnadottir et al. 2010, Caglar et al. 2011; Søvik et al. 2014; Zhang et al. 2014; Ab Halim et al. 2018). On one hand, this is a favorable observation, but the results obtained in the older age group of 18-year-olds, examined in one of the Oral Health Monitoring editions, indicate a very fast progression of lesions (Strużycka et al. 2017). A similar trend was observed in other studies (Dugmore et al. 2004; El Aidi et al. 2008; Bardolia et al. 2010, Kreulen et al. 2010; Hasselkvist et al. 2016; Brusius et al. 2017; Schlueter et al. 2018). This indicates the need to monitor the oral health of children and adolescents not only in terms of the development of caries, but also for non-carious lesions, including erosive lesions.

Gender differences in the incidence of erosive wear, showing higher prevalence and severity in males than in females, have been reported previously (Harding et al. 2003; Larsen et al. 2005; Mulic et al. 2013; Alve et al. 2015; Stenhagen et al. 2017). In our study, ETW was also more prevalent in boys, which was particularly evident in relation to the more advanced lesions.

The socio-economic status is considered an important factor determining the state of health through, among others, eating and hygiene habits; however, the hitherto published research results often contain conflicting information (Al-Dlaigan 2001; Gurgel et al. 2011; Slovic et al. 2014; Skalsky et al. 2018). The adolescents in our study came from different backgrounds, but it is difficult to conclude on the basis of their parents' form of employment what their material status was. We assume that the lack of parental employment and/or part-time employment corresponds to the lower socio-economic status in general. In this group of children, we found more frequent occurrence and higher intensity of ETW. According to Handing and Dugmore’s observations, a change of job to a less qualified one or lack of employment, identified as a decrease in socio-economic status, influences the occurrence of ETW (Dugmore 2003, Harding 2003).

The systemic transformations in Poland had a significant impact on the oral health of the population. The general availability of a wide range of hygiene products, the development of the private dental care sector, an increase in health awareness, but also the collapse of institutional dental care for children and adolescents and, finally, relatively low expenditures on the medical sector create a diversified environment that is difficult to assess unequivocally. The studies carried out in recent years have shown that, with regard to tooth decay, the health of children and adolescents from families with a higher socio-economic status is better compared to their peers from families with lower incomes (Szmidt et al. 2013; Olczak-Kowalczyk et al. 2017). In our own research such the relationship was also observed with regard to ETW. Undoubtedly, a better understanding of the interactions between social, economic and behavioral factors influencing the development of erosive lesions is necessary for the implementation of the correct prophylactic and therapeutic procedures. In our own research, the main problem was the low level of knowledge of the examined adolescents, which could correspond to a similar lack of knowledge in their parents and guardians, not covered by this study.

A balanced diet that meets the current dietary recommendations includes significant intake of vegetables, fruits and juices. However, so-called healthy eating habits may not guarantee an adequate diet. Carbonated, isotonic and energy beverages as well as fruit juices are increasingly becoming an integral part of everyday meals. In Poland, more than 50% of the non-alcoholic beverages market is water, and only 30% is carbonated beverages, but this ratio is changing for the worse. Such eating habits are believed to be responsible for the increased incidence of erosive lesions in many populations of highly developed countries. The conducted meta-analysis showed that the products with the greatest erosive potential include soft beverages, especially those containing vitamin C (Haifeng et al. 2012; Lussi et al. 2012). In our research, carbonated and energy beverages correlated with the erosive lesions in the anterior teeth. The consumption of these beverages was also significantly higher among boys, that might explain the differences in ETW prevalence between boys and girls in the presented study. Other authors published similar results (Lussi et al. 2004; Hasselkvist et al. 2010; Chrysanthakopoulos 2012; Broughton et al. 2016; Margaritis et al. 2019). It will be interesting to observe the changes following the introduction of the Ministry of Health regulation limiting the sale of sweetened and energy beverages in Polish tuck shops in schools.

As mentioned earlier, the level of adolescents’ knowledge on the etiology of erosive wear was unsatisfactory. Most of the study participants did not know about the destructive effects of dietary acids. Similar research results were published by Chłapowska et al. who, having examined 226 students of a Polish medical university, found the need to spread the knowledge about the etiology of tooth erosion to minimize the risk of its effects in the population of young adults (2012). Oral health education is essential to help the society to understand the harmful effects of erosive wear (Chu et al. 2010; Verploegen et al. 2019).

It is necessary to promote dental health education among young people, to increase their motivation to bring about a change in lifestyle, as well as to raise their level of health awareness. It seems that, apart from the acidic diet, it is insufficient information provided to the patients that results in forming bad habits responsible for the development of non-carious teeth defects in adolescents.

The current study has its limitations. Financial and human resources have restricted the study scope to nine provinces. However, it covered all macroregions of the country, and the stratified sampling was used to make sure the results can be considered representative for the entire 15-year-olds population.

The results of self-administered questionnaire are subject to error due to bias, anonymity, and finally current emotional state and physical condition. In addition, the survey was not directed to parents and guardians of adolescents, which could shed light on the state of knowledge in the community they come from as well as provide more details on their economic status.

## Conclusions

Considering the limitations of the resent study the following conclusions can be made:

- The erosive tooth wear (ETW) is a prevalent problem in the population of 15-year-olds in Poland, affecting over 24% of adolescents, especially boys and those of lower socio-economic background.
- While the majority of cases are mild, about 3% of youth present moderate or even severe ETW, especially in the anterior region.
- The severity of the lesions has proven to be related to the carbonated and energy drinks consumption, that is also higher among boys in the studied population.
- Only 12.2% of the study participants were aware of the impact of acidic foods and drinks on the enamel, which can be considered highly unsatisfactory. The lack of knowledge can result in inadequate behaviors, in particular related to diet.
- Given the acceleration of ETW lesions development in young adults, as observed in other studies, our results prompt the introduction of preventive measures, in particular educational, in adolescents.

## Declarations

### Data availability

The datasets used and/or analyzed during the current study are available from the corresponding author on reasonable request.
